# Supplementary material for: Crawling and Gliding: A Computational Model for Shape-Driven Cell Migration
Source: PLoS Comput Biol. 2015 Oct 21;11(10):e1004280. doi: 10.1371/journal.pcbi.1004280 (PMC4619082; doi:10.1371/journal.pcbi.1004280)
Supplement: S1 Code — (ZIP) [file pcbi.1004280.s012.zip › release/tst/doc/html/crash_8h_source.html]

Tissue Simulation Toolkit: crash.h Source File


Tissue Simulation Toolkit
 0.1.4.1


- Main Page
- Namespaces
- Classes
- Files

- File List
- File Members

crash.h

Go to the documentation of this file.

1 /\*

2

3 Copyright 1996-2006 Roeland Merks

4

5 This file is part of Tissue Simulation Toolkit.

6

7 Tissue Simulation Toolkit is free software; you can redistribute

8 it and/or modify it under the terms of the GNU General Public

9 License as published by the Free Software Foundation; either

10 version 2 of the License, or (at your option) any later version.

11

12 Tissue Simulation Toolkit is distributed in the hope that it will

13 be useful, but WITHOUT ANY WARRANTY; without even the implied

14 warranty of MERCHANTABILITY or FITNESS FOR A PARTICULAR PURPOSE.

15 See the GNU General Public License for more details.

16

17 You should have received a copy of the GNU General Public License

18 along with Tissue Simulation Toolkit; if not, write to the Free

19 Software Foundation, Inc., 51 Franklin St, Fifth Floor, Boston, MA

20 02110-1301 USA

21

22 \*/

23 void StartSIGINTHandling();

24 void HandleSIGINT(int dummy);

25 void StartSIGSEGVHandling();

26 void HandleSIGSEGV(int dummy);

27 void NiceMessage();

28 void MemoryWarning(void);

29 void Crash(char \*message);

MemoryWarning

void MemoryWarning(void)

**Definition:** crash.cpp:85

HandleSIGSEGV

void HandleSIGSEGV(int dummy)

**Definition:** crash.cpp:62

StartSIGSEGVHandling

void StartSIGSEGVHandling()

**Definition:** crash.cpp:54

HandleSIGINT

void HandleSIGINT(int dummy)

**Definition:** crash.cpp:48

NiceMessage

void NiceMessage()

**Definition:** crash.cpp:77

Crash

void Crash(char \*message)

**Definition:** crash.cpp:94

StartSIGINTHandling

void StartSIGINTHandling()

**Definition:** crash.cpp:41


---

Generated on Thu Aug 14 2014 22:04:01 for Tissue Simulation Toolkit by  

 1.8.6
